# Development of implicit and explicit attentional modulation of the processing of social cues conveyed by faces and bodies in children and adolescents

**DOI:** 10.3389/fpsyg.2023.1320923

**Published:** 2023-12-27

**Authors:** Viola Oldrati, Alessandra Bardoni, Geraldina Poggi, Cosimo Urgesi

**Affiliations:** ^1^Scientific Institute, IRCCS E. Medea, Bosisio Parini, Lecco, Italy; ^2^Laboratory of Cognitive Neuroscience, Department of Languages and Literatures, Communication, Education and Society, University of Udine, Udine, Italy

**Keywords:** attention, children, adolescence, face processing, body processing, emotion, sex

## Abstract

Emotions and sex of other people shape the way we interact in social environments. The influence of these dimensions on cognitive processing is recognized as a highly conditional phenomenon. While much of researches on the topic focused on adults, less evidence is available for the pediatric population. This study aimed at examining the development of the modulation of attention control on emotion and sex processing using facial and body expressions in children and adolescents (8–16 years old). In Experiment 1a, participants performed a Flanker task (probing space-based attention) in which they had to indicate either the emotion (happy/fearful) or the sex of the target stimulus while ignoring the distracting stimuli at the side. We found evidence for intrusion of the sex, but not emotion, of the stimuli during both sex and emotion recognition tasks, thus both at an explicit (i.e., task relevant) and implicit (i.e., task irrelevant) level. A control experiment consisting of an emotional Flanker task confirmed that, in contrast with previous findings in adults, emotion did not modulate attention control in children and adolescents even when task relevant (Experiment 1b). In Experiment 2 participants performed a same-or-different judgment task (probing feature-based attention) in which they indicated whether the central stimulus matched the lateral ones for emotion or sex. Results showed that emotional features exerted an implicit influence during sex judgements; likewise, sex features intruded on the processing of both faces and bodies during emotion judgments. Finally, Experiment 3 explored the development of the explicit attention modulation exerted by the sex dimension on the processing of faces and bodies. To this aim, participants performed a Flanker task in which they were asked to recognize the sex of faces and bodies. The results indicated that, while younger participants showed a task-relevant influence of sexual features when processing faces, older participants showed such influence when processing bodies. These findings point to a greater attentional modulation exerted by sex, as compared to emotion, during social processing in children and adolescents and suggest a developmental trend of the saliency of facial and bodily cues for the perception of others’ sex.

## Introduction

Maintaining the focus of attention on an ongoing task while also diverting it to significant incoming cues is crucial for adapting to a complex, changing environment. While proactive attention mechanisms, often referred to as top-down processes, are employed to sustain mental resources on ongoing tasks, reactive bottom-up processes are triggered by stimuli that possess novelty or salience. For instance, facial and bodily cues, such as emotion expressions and sexually dimorphic cues, may influence cognitive processing by shaping our perceptions, attitudes, and decision-making processes without conscious awareness. Extensive lines of research supported the idea that emotionally-salient stimuli benefit of a pre-attentive processing compared to neutral or non-emotional stimuli, due to their evolutionary significance (Öhman et al., 2000). Given their importance in shaping social interactions, previous studies on the relationship between emotion processing and attention control focused on facial (and, less extensively, bodily) emotion expressions. Faster and/or more accurate responses, suggesting a more automatic processing for emotional expressions, have been reported in detecting angry/frightened faces amidst or compared to neutral or happy faces (Fox et al., 2000; Lundqvist and Öhman, 2005; Schubö et al., 2006; Lobue, 2009). Even so, other findings failed to observe differences in some index of performance in response to emotional and non-emotional face expressions (Schulz et al., 2009).

However, many other findings indicated that directing attention to emotional expressions (regardless of their valence) is not automatic, but it is contingent on how relevant the emotional aspect is to the goals of the ongoing task (Barratt and Bundesen, 2012; Victeur et al., 2020; Calbi et al., 2022; Mancini et al., 2022; Mirabella et al., 2023). Whether the processing of emotional cues may benefit from pre-attentive processing or is susceptible to the modulation of top-down mechanisms, it likely depends on the relevance of the stimuli within a given context, i.e., the specific contingencies of the environment, the individual and his/her goals, in line with the insights provided by the appraisal theories of emotion (Moors and Fischer, 2019). Furthermore, other aspects, such as arousal and stimulus complexity (often overlooked), have been identified as confounding variables concurring to explain the influence of emotional cues at an implicit level, namely, even when they are not relevant to the task at hand (Calbi et al., 2022). Neuroimaging evidence also supports that attention control does modulate emotion processing. The attentional competition triggered by the prioritization of task-related stimulus (through top-down mechanisms) and the automatic capture of stimulus saliency (through bottom-up mechanisms) is believed to engage distinct but complementary neural circuits. Functional magnetic resonance imaging (fMRI) studies have demonstrated that amygdala response to emotional versus neutral stimuli decreases as the cognitive demands of the primary task increase (Pessoa et al., 2005, 2006) or when the emotional stimuli are not consciously attended to (Vuilleumier et al., 2002).

Many of the cited studies compared different emotions to each other (e.g., happy vs. fearful faces) or, when presenting emotional faces as distractors, they required participants to focus their attention on a completely different type of stimuli (e.g., houses) or non-social feature (e.g., color of the faces). In this regard, comparing facial and bodily emotional expressions with their sexual features may represent a more ecological approach (see Zhou and Liu, 2013; Zhou et al., 2016; Oldrati et al., 2020; Mancini et al., 2022; Mirabella et al., 2023; Montalti and Mirabella, 2023). Indeed, the emotional and sexual features of faces and bodies represent crucial information for adapting in complex social environments. Accordingly, the human visual system displays a strong sensitivity to both emotional (Meeren et al., 2005) and sexual features (Bruce et al., 1993; Johnson and Tassinary, 2005), conveyed by either of faces or bodies.

In a study applying a Flanker paradigm (Zhou and Liu, 2013), the researchers asked participants to indicate the emotion or the sex of a computer-generated target face, which could either match or not two flanker stimuli for emotion or sex. In the emotion recognition task, the emotional features were task-relevant and the sexual ones were task-irrelevant; conversely, in the sex recognition task, the sexual features were task-relevant and the emotional ones were task-irrelevant (Zhou and Liu, 2013). They found that the incongruence of emotional (or sexual) features between the target and the flankers interfered with the discrimination of emotion (or sex) when task-relevant but not when task-irrelevant, suggesting the modulation of top–down regulation mechanisms on both dimensions. By coupling the same paradigm with ERPs recording (Zhou et al., 2016), the researchers observed that the emotional conflict was more pronounced when task-relevant than when task-irrelevant; yet, the conflict, signaled by the cortical component N200, rose earlier for emotional than for gender incongruence. In light of this evidence, the authors concluded that attentional control might impact emotion processing, but also that the saliency of emotional information may be more susceptible to bottom-up processing, resulting in comparatively lesser top-down modulation compared to non-emotional stimuli (such as sexual cues).

In the wake of these last two studies, we recently conducted a study aimed at examining the modulatory effect of attention on emotion and sex processing when conveyed by faces and bodies, adopting a similar Flanker task (Oldrati et al., 2020). The results showed that emotional features, but not sexual ones, modulated the processing of both face and body targets when either task-relevant (during emotion recognition) or task-irrelevant (during sex recognition). Interestingly, in another experiment within the same study, where participants were asked to match the emotion or the sex of the central and lateral stimuli, we observed a bottom-up (task-irrelevant) intrusion of the emotional dimension on the comparison of sexual features of bodies, but not faces, perhaps due to the specific role of bodies in the communication of emotions (see Oldrati et al., 2020 for a discussion). Taken together, these findings aligned to studies indicating that emotional stimuli may benefit to some extent from a prioritized, pre-attentive processing (Carretié et al., 2009; Rellecke et al., 2011; Junhong et al., 2013).

While much of the research on the interplay between emotion processing and attention control focused on adults, less evidence is available for the pediatric population. However, it is known that cognition in developmental age is susceptible to competing information (Bunge et al., 2002; Durston et al., 2002), especially for emotionally salient information. Focusing on earlier stages of life, a study reported that 8- to 14-month-old infants oriented their gaze more rapidly to angry than to happy faces (Lobue and Deloache, 2010). Comparable results were observed using the same task in 5-years old children (Lobue, 2009). Older children (aged 8-12-years old) showed an attentional bias for happy faces as compared to neutral ones in a visual-probe task, while a bias for angry faces was observed exclusively in highly anxious children (Waters et al., 2010). Conversely, Zamora et al. (2020) by administering a perceptual (color) Flanker task to 8-12-years old children, found that irrelevant distractors (including images of faces, animals, objects, and landscapes appearing around the Flanker array) affected performance; however, there were no differences according to whether these distractors were emotionally salient or neutral. Réveillon et al. (2020) administered a Flanker task in which the array was composed of images of faces (happy, fearful or neutral) or scrambled images, but they asked children (aged 9–11 years old) to respond according to the color of the stimulus; thus, the emotion expression was a task-irrelevant feature. Similar to Zamora et al. (2020), they found that incongruent trials elicited slower responses than congruent ones for face but not for scrambled-image arrays, highlighting the saliency of social-cues. However, no differences were detected according to whether faces were emotional or neutral. Concerning adolescents, a recent study observed an interference of incongruent trials leading to slower response in both non-emotional flankers and flankers with superimposed emotional faces in 13-17-years old participants relative to young adults (Mo et al., 2023). Overall, the conclusion that top–down and bottom–up mechanisms in the processing of emotion processing mutually influence each other in a highly conditional way appears to extend from adults to children and adolescents. Moreover, the inconsistency in findings, either supporting or challenging the modulation of attention on emotion processing, could reflect methodological differences across studies. Indeed, previous studies, in either adult or pediatric age, applied a broad variety of experimental paradigms (including, for example, go/no-go, Stroop or Flanker paradigm), outcome measures (either behavioral and/or neural) and type of stimuli (e.g., static or dynamic, photographs or computer-generated images).

The present work was aimed at examining the interplay between emotion processing and attention control in a sample of children and adolescents. To allow a clear comparison with adult performance, we maintained the same procedure and task structure as in our previous work on adults (Oldrati et al., 2020). This choice was made to enhance methodological consistency and facilitate a discussion on potential age-related differences and trajectories of development. Furthermore, unlike most of the research on the topic, the selected tasks required to focus alternatively on either the emotion or the sex of facial or bodily stimuli. As mentioned above, these two dimensions are intrinsic features of both faces and bodies and, thus, their comparison may represent an ecological approach. Since here participants were asked to label the facial/bodily stimuli as female or male based on the secondary sexual characteristics, we decided to use the term “sex,” in line with current guidance.^1^

First, we administered a modified Flanker task to test whether the processing of emotion or sex information is modulated by attentional control when task-relevant, or whether it interferes with the ongoing task when task-irrelevant. Then, we tested whether the same effects were maintained when using a same-or-different comparison task. This second paradigm allowed us to explore the modulatory role of distinct visual search strategies on stimuli processing and distractor filtering (Maunsell and Treue, 2006). While the Flanker task invites to focus the attention on the central target of the array, which may favor space-based filtering of distractors, the same-or-different judgment task requires to expand the focus of attention to the entire array. This could discourage space-based filtering (i.e., suppressing the processing of an object based on its location) and favor a feature-based strategy (i.e., suppressing the processing of an image component related to a particular feature).

Another aim was to explore the modulatory effect of attention on these social cues conveyed not only by faces, but also by bodies. In fact, both faces and bodies, alongside providing important information for the adaptation to social environments, share similar cognitive mechanisms and neural correlates (see Minnebusch and Daum, 2009 for a detailed review).

### Experiment 1a

We administered a Flanker paradigm to test whether the attentional modulation (i.e., task relevance) of the relative influence of sex and emotional cues was comparable for face and body stimuli. A central face or body target was presented on a screen sided by two flanker stimuli, respectively faces or bodies, that could match or not the central target in emotion or sex. In separate blocks, participants were required to discriminate the emotion (i.e., positive or negative) or the sex (i.e., male or female) of the central target, while trying to ignore the flanker stimuli. Based on what observed in adults (Oldrati et al., 2020), we expected emotional, but not sexual features, to influence the performance when both task-relevant (during emotion recognition) and task-irrelevant (during sex recognition). Furthermore, we expected to observe this effect in relation to both faces and bodies.

#### Participants

Twenty-four typically developing children and adolescents (8 M / 16F, age range = 8–16 years old, *M* = 11.5, SD = 2.6) participated in Experiment 1a. In more detail, the sample included four 8-years old, two 9-years old, four 10-years old, one 11-years old, five 12-years old, two 13-years old, three 14-years old and three 16-years old participants.

The sample size was based on the previous study applying the same procedure and tasks in adults (Oldrati et al., 2020). The following information applies to all the experiments of the study. Participants were enrolled in local schools and tested individually in the school setting by an experimenter, outside the academic hours. All participants had normal or corrected-to-normal vision. Prior to the beginning of the experiment, written informed consent was obtained from all legal guardians of the participants. Participants provided their verbal consent. The study was approved by the local ethical committee (protocol: CGPER-2021-04-12-01) and conducted in accordance with the declaration of Helsinki.

#### Stimuli and task

Experimental stimuli and task structure were identical to those used in a previous experiment testing the very same hypothesis in a sample of healthy adults (Oldrati et al., 2020). Stimuli consisted of a total of 24 pictures of Caucasian faces and bodies. Images of faces were taken from the NimStim dataset (Tottenham et al., 2009). Previous work demonstrated that the NimStim pictures of adult actors is sufficiently reliable for use with 2-6-years old children who were typically developing (Barnard-Brak et al., 2017). Two emotion expressions (happy and fearful) were chosen, so that in total six female and six male faces (three happy and three fearful) were used (6 identities in total). Images of bodies were taken from a validated pool of static images depicting bodies with blurred faces in emotional whole-body movements (see Borgomaneri et al., 2012 for details). As for faces, two emotion expressions (happy and fearful) were chosen, so that in total six female and six male bodies (three happy and three fearful) were used in the task (6 identities in total).

**Figure 1:**
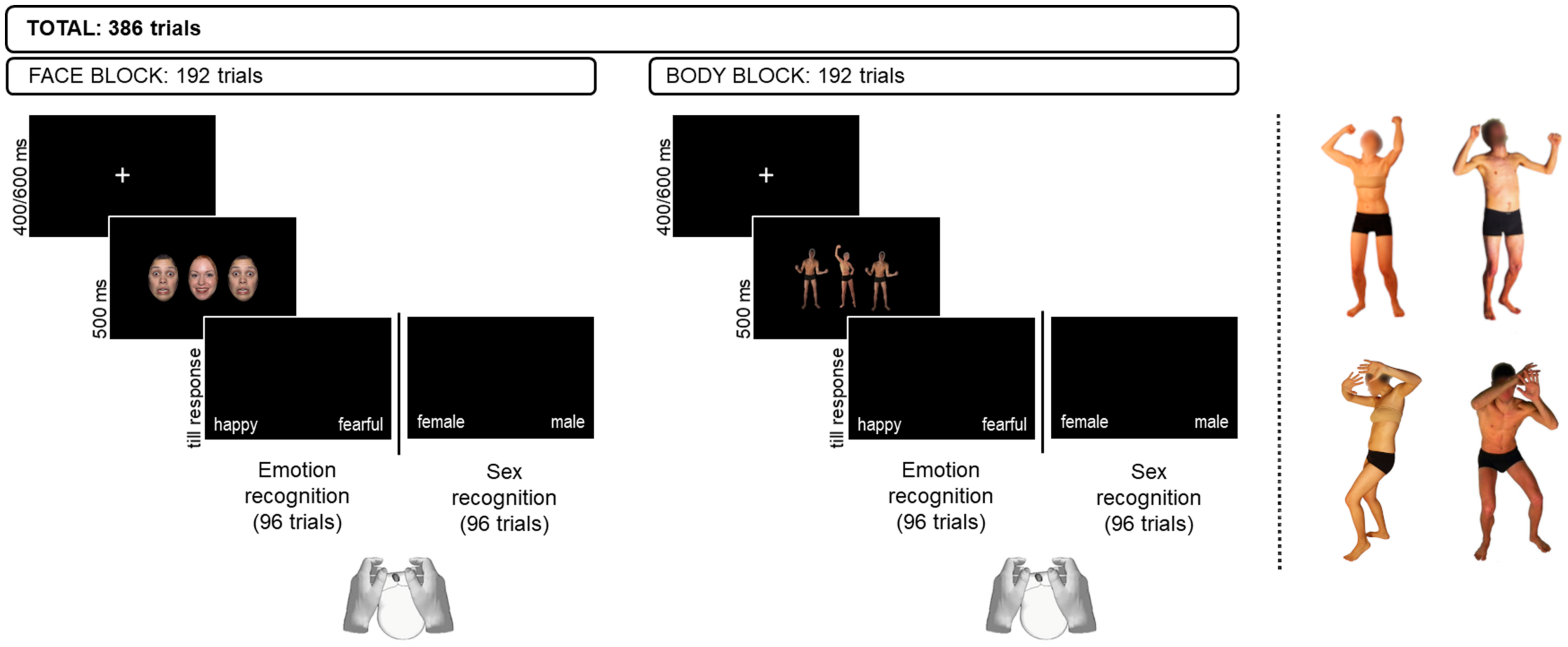
Example of experimental trials displaying faces (left panel) and bodies (right panel). In the example, the face array represents an incongruent trial in the Emotion recognition task and a congruent one in the Sex recognition task. Conversely, the body array represents a congruent trial in the Emotion recognition task and an incongruent trial in the Sex recognition task. At the right of the dashed line are reported examples of happy (top) and fearful (bottom) body postures of female (left) and male (right) actors. Original faces used in the study available from https://danlab.psychology.columbia.edu/content/nimstim-set-facial-expressions upon request. Body images reproduced with permission from Borgomaneri et al. 2012.

Participants were presented with two Flanker tasks (Eriksen and Eriksen, 1974) requiring to focus either on the emotion expression or on the sex of the stimuli. Face and body stimuli were presented in separate blocks. Face and body stimuli were displayed on a laptop screen, with participants sitting approximately 60 cm from the screen. Each trial displayed an array of three stimuli on a dark background. A visual angle of 3° wat set between the center of the target and the center of each flanker. Participants were asked to indicate the emotion (emotion recognition task) or the sex (sex recognition task) of the face/body displayed in the middle of the array (i.e., the target) while ignoring the ones displayed at the side (i.e., the flanker). Emotional and sex features across the array of stimuli were presented in four different combinations: different emotion / different sex, different emotion/ same sex, same emotion / different sex, same emotion / same sex. In the emotion task, emotional features were task-relevant and sex features were task-irrelevant, whereas in the sex task, emotional features were task-irrelevant and sex features were task-relevant. Face and body arrays were presented in separate blocks, whose order was counterbalanced among participants. Similarly, the order of presentation of the two tasks within each stimulus type block was counterbalanced between participants. Participants provided their response with a left/right mouse click using their thumbs. Response key assignment was counterbalanced among participants. The array of stimuli appeared on the screen for 500 ms, followed by a blank screen which was kept until the participant had provided his/her response. In keeping with a similar study on children (Zamora et al., 2020), we did not set a time limit for the participants to respond, but we invited them to respond as quickly and accurately as possible. A white fixation cross of 400–600 ms appearing in the center of the screen was presented between stimuli presentation. Accuracy and reaction times (RTs) were automatically recorded for offline analysis. Figure 1 provides a schematic depiction of experimental trials and reports the number of trials per block (face vs. body) and task (emotion vs. sex recognition). The software E-prime 3.0 (Psychology Software Tools, Pittsburgh, PA) was used for stimulus presentation and data collection for all experiments.

#### Data handling and statistical analysis

To begin with, data were filtered so that trials with latencies falling below 150 ms and above 4,000 ms from the stimulus onset were removed from the final dataset. This filtering was applied to the data of all the experiments of the study.

For data collected in Experiment 1, a Binomial Generalized Linear Mixed Model (GLMMs) was estimated for raw accuracy and a Restricted Maximum Likelihood (REML) - fitted LMMs was estimated for RTs of correct responses. The models included TASK (emotion recognition vs. sex recognition), STIMULUS-TYPE (face vs. body), EMOTION CONGRUENCY (congruent vs. incongruent emotion) and SEX CONGRUENCY (congruent vs. incongruent sex) and their 4-way interaction as fixed factors, their intercepts, SUBJECTS and STIMULUS IDENTITY (of the target) as random factors. For RTs analysis, only trials in which the participants provided a correct response were included in the models. For this and all the following experiments, statistical significance was obtained by a type II Wald chi-square. Post-hoc comparisons were carried out applying false discovery rate (FDR) adjustment. An advantage of LMMs is that they allow accounting for random differences in outcome values between participants and/or between items or conditions characteristics (Meteyard and Davies, 2020). All data are reported as Mean (M) ± Standard Error of the mean (SE). All the models were estimated using R (version 4.0.4) and lmerTest R package (Kuznetsova et al., 2017).

In addition, Bayesian Factor (BF) were computed to determine whether expected effects yielding non-significant results supported a null hypothesis of such effects (Schmalz et al., 2021). To this aim, we used the BayesFactor R package (Morey and Rouder, 2022), keeping the default priors (r = 0.707).

#### Results on accuracy

**Figure 2:**
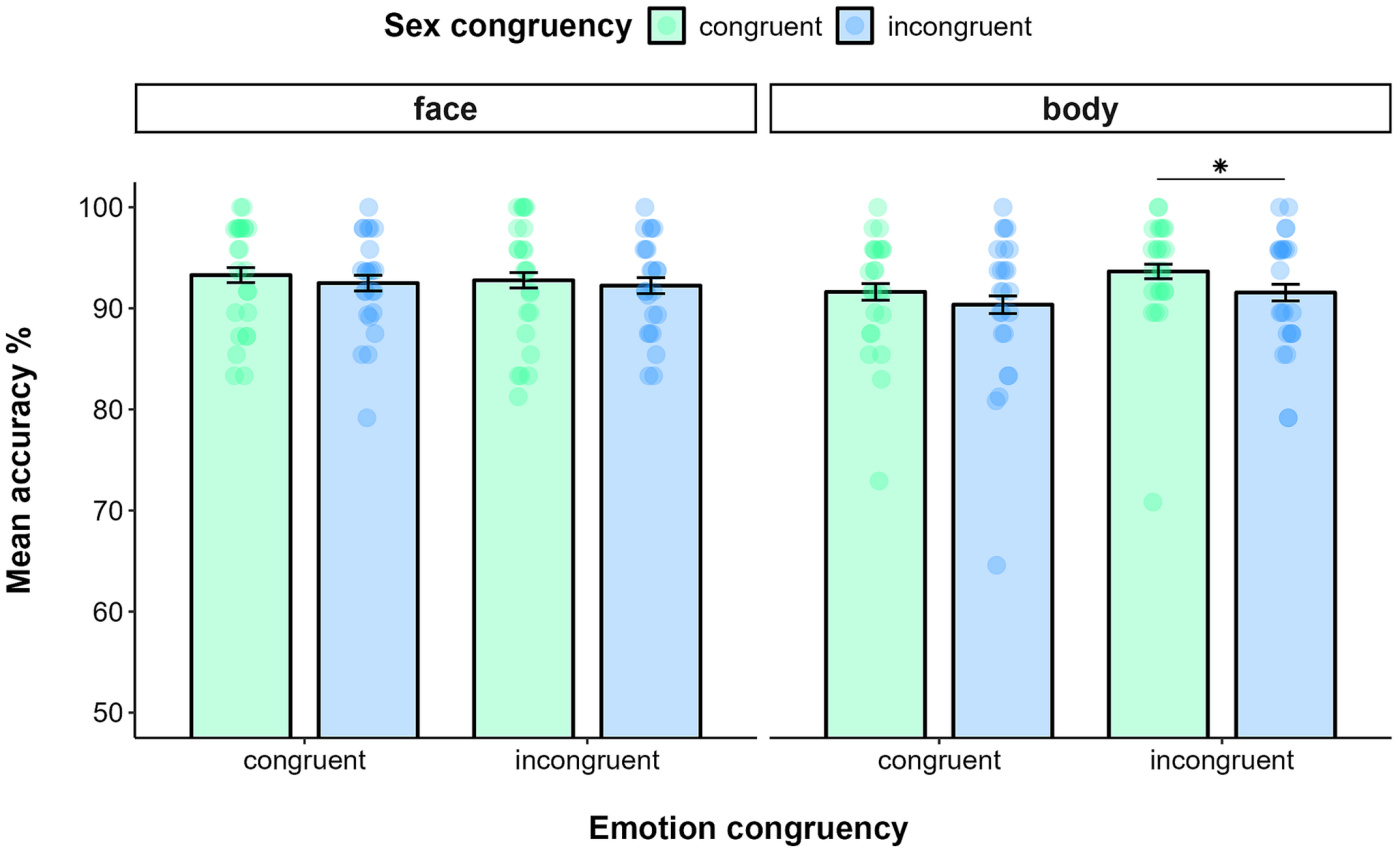
Mean accuracy in the Emotion recognition task as a function of stimulus type (face vs. body), Emotion congruency (on the x-axis) and Sex congruency in Experiment 1a. Error bars represent ±1 SE. * *p* < 0.05.

Data filtering resulted in deleting 0.43% of trials in the emotion recognition task and 0.22% of trials in the sex recognition task. The GLMM on accuracy yielded a main effect of TASK (χ_(1)_ = 44.24, *p* < 0.0001), as participants were more accurate in recognizing the sex (*M* = 0.94, SE = 0.01) than the emotion expression of the target (*M* = 0.90, SE = 0.01). The analysis also showed a significant main effect of SEX CONGRUENCY (χ_(1)_ = 4.39, *p* = 0.04), indicating that participants, regardless of the task at hand, were more accurate in recognizing the target emotion (or sex) in sex congruent trials (*M* = 0.93, SE = 0.01) than in sex incongruent ones (*M* = 0.92, SE = 0.01). From the analysis emerged a significant interaction effect of TASK*STIMULUS-TYPE (χ_(1)_ = 6.47, *p* = 0.01). Post-hoc analysis showed that participants were more accurate in recognizing the sex of faces (*M* = 0.95, SE = 0.01) than that of bodies (*M* = 0.92, SE = 0.01, *p* = 0.03), whereas they were comparably accurate in recognizing the emotion of faces (*M* = 0.90, SE = 0.01) and bodies (*M* = 0.91, SE = 0.01, *p* = 0.69). The interaction effect of TASK*STIMULUS-TYPE*SEX CONGRUENCY was also significant (χ_(1)_ = 6.66, *p* = 0.01). Indeed, participants were more accurate in recognizing the emotion of bodies (i.e., in the emotion recognition task) in sex congruent trials (*M* = 0.92, SE = 0.01) than in sex incongruent trials (*M* = 0.89, SE = 0.01; *p* = 0.01), whereas such a difference did not emerge for faces (sex congruent trials: *M* = 0.90, SE = 0.01; sex incongruent trials: *M* = 0.90, SE = 0.01; *p* = 0.91). Nor did we detect any difference between congruent and incongruent trials when participants were asked to recognize the sex of either target faces (i.e., in the sex recognition task; sex congruent trials: *M* = 0.96, SE = 0.01; sex incongruent trials: *M* = 0.94, SE = 0.01; *p* = 0.15) or target bodies (sex congruent trials: *M* = 0.93, SE = 0.01; sex incongruent trials: *M* = 0.93, SE = 0.01; *p* = 0.72). This effect was further specified by the significant four-way interaction of TASK* STIMULUS-TYPE* EMOTION CONGRUENCY* SEX CONGRUENCY (χ_(1)_ = 4.48, p = 0.03). The effect was explained by the fact that, only in the emotion recognition task and when the emotion of target and flankers was incongruent, participants were more accurate in recognizing the emotion of the target body when the sex across target and flanker was congruent (*M* = 0.94, SE = 0.02) compared to when it was incongruent (*M* = 0.89, SE = 0.02; p = 0.01) (Figure 2). No other interaction was significant (all *p* > 0.1).

The BF of the hypothesis of an effect of EMOTION CONGRUENCY, which was expected but disconfirmed by the frequentist analysis, was calculated in two repeated-measures ANOVAs (rm-ANOVAs) separately for the emotion recognition and the sex recognition task, with the within-subject factors STIMULU-TYPE and EMOTION CONGRUENCY. For the emotion-recognition model, the (task-relevant) effect of EMOTION CONGRUENCY yielded a BF_10_ = 0.17, providing moderated evidence for the null hypothesis. The interaction effect of EMOTION CONGRUENCY*STIMULUS-TYPE yielded a BF_10_ = 0.45, providing anecdotal evidence for the null hypothesis of the interaction. For the sex-recognition model, the (task-irrelevant) effect of EMOTION CONGRUENCY yielded a BF_10_ = 0.27, providing moderated evidence for the null hypothesis. The interaction effect of EMOTION CONGRUENCY*STIMULUS-TYPE yielded a BF_10_ = 0.46, providing anecdotal evidence for the null hypothesis of the interaction.

#### Results on RTs

The analysis on RTs yielded a significant effect of TASK (χ_(1)_ = 131.47, *p* < 0.0001), indicating that participants were faster in recognizing the sex (*M* = 849.13, SE = 34.19) than the emotion (*M* = 933.88, SE = 35.49) of the target stimuli. No other main or interaction effects were significant (all *p* > 0.1).

The BF of the hypothesis of an effect of EMOTION CONGRUENCY was calculated following the procedure and model specifications described above. For the emotion-recognition model, both the (task-relevant) effect of EMOTION CONGRUENCY (BF_10_ = 0.17) and the interaction effect of EMOTION CONGRUENCY*STIMULUS-TYPE (BF_10_ = 0.24), provided moderated evidence for the null hypothesis. For the sex-recognition model, both the (task-irrelevant) effect of EMOTION CONGRUENCY (BF_10_ = 0.15) and the interaction effect of EMOTION CONGRUENCY*STIMULUS-TYPE (BF_10_ = 0.27), provided moderated evidence for the null hypothesis.

Accuracy and RTs among conditions in both tasks are reported in Table 1.

**Table 1:** Mean (SD) RTs and Accuracy for each experimental condition in Experiment 1a.

		Emotion recognition				Sex recognition			
		Face		Body		Face		Body	
Emotional features	Sexual features	RTs	Acc	RTs	Acc	RTs	Acc	RTs	Acc
*Congruent*	*Congruent*	945.3 (205.4)	0.91 (0.08)	931.1 (205.6)	0.91 (0.06)	848.4 (196.5)	0.96 (0.05)	834.0 (170.5)	0.93 (0.08)
	*Incongruent*	945.1 (220.2)	0.90 (0.09)	927.6 (188.8)	0.89 (0.10)	853.4 (196.7)	0.95 (0.05)	856.8 (196.7)	0.92 (0.07)
*Incongruent*	*Congruent*	935.0 (211.1)	0.89 (0.10)	943.6 (187.3)	0.94 (0.05)	856.7 (141.9)	0.96 (0.05)	840.3 (181.6)	0.93 (0.09)
	*Incongruent*	931.1 (197.9)	0.90 (0.07)	908.4 (163.4)	0.89 (0.08)	860.4 (184.4)	0.94 (0.05)	838.6 (193.5)	0.95 (0.06)

#### Discussion experiment 1a

In this Flanker task, participants were asked to recognize either the emotion (positive/negative) or the sex of the target stimulus while ignoring the distractors, which could match or not either the task-relevant features (e.g., emotional features in emotion discrimination) or the task-irrelevant ones (e.g., sex features in emotion discrimination). The results suggested that, exclusively in the Emotion recognition task displaying bodies, the congruency between the sex of the target and of the flankers impacted the emotion discrimination of the target bodies in emotion incongruent trials. Put in other words, participants were more accurate in recognizing the emotion when the sex of the target matched the sex of the flanker (sex congruency), but only in trials in which the emotion of the target did not match the emotion of the flanker (emotion incongruence). This result indicated the presence of an interaction between emotion and sex. Indeed, the interdependency between these two dimensions can be appreciated in how changes in one aspect, namely sex, disrupted the recognition of the other aspect, namely emotion.

Evidence of the so-called Garner interference effect points to the challenge faced by the attentional control system in disregarding sexual or emotional cues when the primary objective is centered around emotion or sex, respectively (Gilboa-Schechtman et al., 2004; Atkinson et al., 2005; Becker, 2017). While most of the research on the topic focused on faces, recently, Craig and Lipp (2023) found that also bodily sex cues moderated emotion categorization and, vice versa, the emotion expression of bodies moderated sex categorization, aligning with previous evidence on face processing. This result conflicts with the absence of Gardner effect previously reported by Gandolfo and Downing (2020). These conflicting findings might be linked to methodological factors, such as the used emotions, the use of a costum-created vs. validated set of stimuli and insufficient power (Craig and Lipp, 2023). The fact that we observed this effect only for body but not for face processing may be owed to the fact that participants displayed an advantage in recognizing the sex of faces than of bodies (see also Discussion of experiment 3).

Contrary to what previously observed in adults (Oldrati et al., 2020), the emotional valence did not modulate the performance neither when task-relevant (during emotion recognition) nor when task-irrelevant (during sex recognition). Rather, the main effect of sex congruency, although in presence of an interaction effect that further specified its impact on performance, suggests that, in children and adolescents, the sex dimension may be more influential than the emotion in guiding the processing of faces and bodies.

To confirm the lack of effect of emotion in modulating attention control at an explicit level, we performed a control experiment (1b) by administering an emotional Flanker task with no variation of the sexual dimension across the stimuli of the array.

### Experiment 1b

We administered an emotional Flanker task to test whether the emotional dimension would modulate attention control at an explicit level. To this aim, a central target was presented on a screen sided by two flanker stimuli that could match or not the central target in emotion. Participants were required to recognize the emotion (i.e., positive or negative) of the central target, while trying to ignore the flanker stimuli. This control experiment was carried out to follow-up on the results of Experiment 1b, which indicated that emotional cues did not influence the recognition of the target when task-relevant. Since the interaction effect emerged only for bodily expressions, this control experiment presented only bodies.

#### Participants

Twenty-four healthy children and adolescents (8 M/16F, age range = 8–16 years old, *M* = 12.42, SD = 2.75) participated in Experiment 1b. In more detail, the sample included one 8-years old, six 9-years old, one 11-years old, four 12-years old, two 13-years old, four 14-years old, one 15-years old and five 16-years old participants. None of the participants enrolled in this experiment participated in Experiment 1a.

#### Stimuli and task

Experimental stimuli and task structure were identical to those used in Experiment 1a. However, here only images of bodies were presented, and the sex of the bodies did not vary within each trial (i.e., all male or all female bodies).

#### Results on accuracy

**Figure 3:**
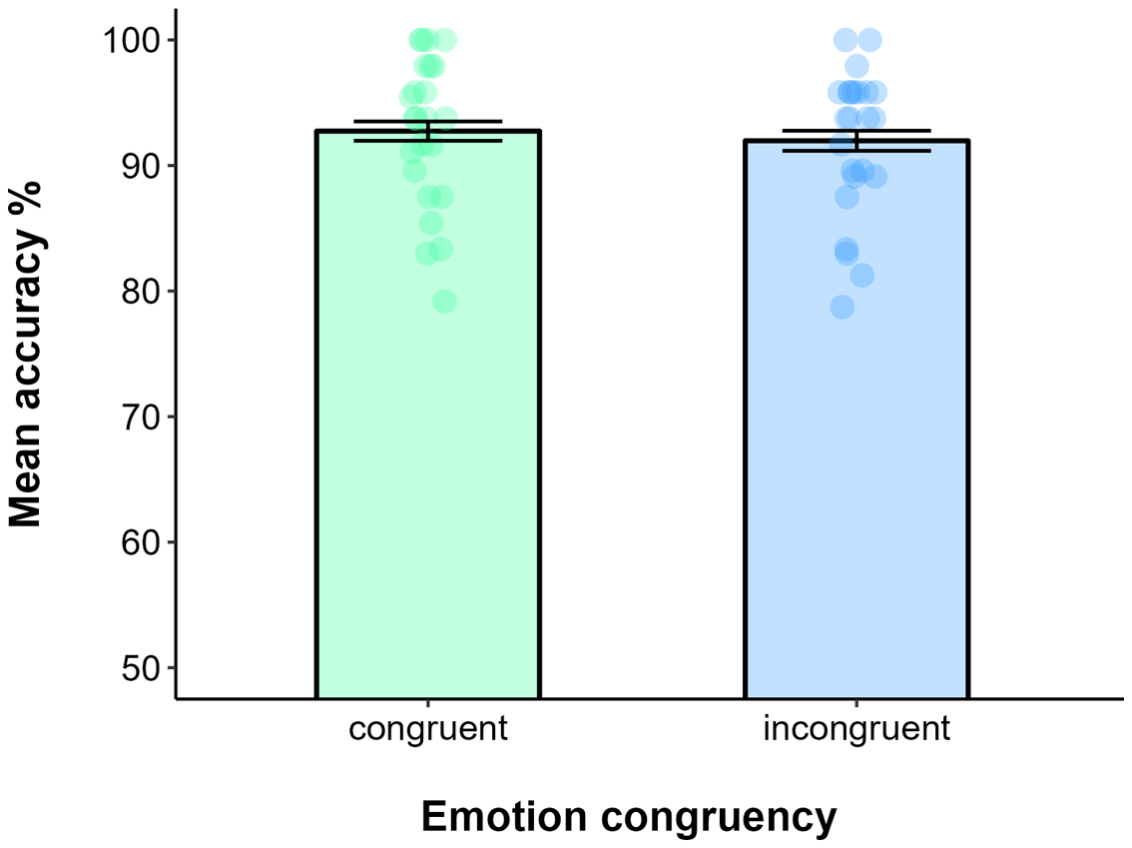
Mean accuracy in the Emotion recognition task of bodily stimuli as a function of Emotion congruency in Experiment 1b. Error bars represent ±1 SE.

Data filtering resulted in deleting 0.61% of trials. The EMOTION CONGRUENCY was the only fixed factor included in the GLMM, while intercepts and SUBJECTS were included as random factors. The model on accuracy showed that the effect of EMOTION CONGRUENCY was not significant (χ_(1)_ = 0.53, *p* = 0.47), as the performance among congruent (*M* = 0.93, SE = 0.01) and incongruent trials (*M* = 0.92, SE = 0.01) was comparable (see Figure 3).

In addition, we carried out exploratory analysis to examine the potential impact of the age of participants on both accuracy and RTs, by adding in the supplementary model the factor AGE and its interaction with the other fixed effects. This analysis was not performed on the data of experiment 1a due to a converge problem of the model. The exploratory analysis examining the potential effect of AGE did not yield any significant effect (all *p* > 0.09), confirming the results of the previous model.

As in experiment 1a, we calculated the BF of the effect of EMOTION CONGRUENCY. The analysis yielded a BF_10_ = 0.57, providing anecdotal evidence for the null hypothesis.

#### Results on RTs

The analysis on RTs also did not yield a significant result effect of EMOTION CONGRUENCY (χ_(1)_ = 0.05, *p* = 0.81; congruent, *M* = 902.18, SE = 29.91; incongruent, M = 899.33, SE = 30.42). The exploratory analysis yielded a significant main effect of AGE (χ_(1)_ = 0.05, p = 0.81): the older the participants, the faster their responses. No other effect was significant (all *p* > 0.3).

Finally, the effect of EMOTION CONGRUENCY yielded a BF_10_ = 0.32, providing moderate evidence for the null hypothesis (see Figure 3).

#### Discussion experiment 1b

The results confirmed that the emotion conveyed by body images did not influence the recognition of the target at an explicit level, as indicated by the absence of an emotion congruency effect on both accuracy and RTs. Together with the result of the previous experiment, indicating an absence of both a task-irrelevant and task-relevant influence of the emotional cues (although in presence of a concomitant variation of sexual cues that was here eliminated), our data indicated that, in our sample of children and adolescents, emotional features did not influence the allocation of attention by spatial-location filtering nor via top-down or bottom-up processes.

Lastly, we found a more pronounced improvement in the processing of bodies, driven by a facilitation of face over body processing in younger participants, which tended to disappear with increasing age (see General Discussion).

### Experiment 2

This experiment was conceived to test whether expanding the focus of attention across the visual field may modulate the interplay between attentional control and emotional and sex feature processing. With this goal, we applied a same-or-different judgment paradigm, in which participants compared the emotion or the sex of central and lateral stimuli, and tested the effects of the task-irrelevant dimension (i.e., sex congruency in the emotion task and emotion congruency in the sex task).

In light of the results of experiment 1b, suggesting a greater saliency of sexual cues than the emotional ones, we expected the sexual dimension to exert a bottom-up interference on the comparison of emotional features. Additionally, since here we increased the relevance of the lateral stimuli by expanding the focus of attention across the stimulus array (i.e., we asked the participants to compare the central and lateral stimuli rather than to simply ignore the lateral ones on the basis of their spatial position), we expected also an intrusion of emotion task-irrelevant features on the processing of faces and bodies at an implicit level.

As in experiment 1b, we carried out exploratory analysis to examine the potential impact of the age of participants on both accuracy and RTs, by adding in the supplementary model the factor AGE and its interaction with the other fixed effects.

#### Participants

Twenty-four healthy children and adolescents (11 M / 13F, age range = 8–16 years old, *M* = 10.8, SD = 2.5) participated in Experiment 2. In more detail, the sample included five 8-years old, four 9-years old, three 10-years old, three 11-years old, two 12-years old, three 13-years old, two 14-years old, one 15 years-old and one 16-years old participants. A Welch corrected t-test indicated that the age of the participants of Experiment 1a and 2 was comparable (t_46_ = 0.96, *p* = 0.34). The distribution of male and female participants was also comparable among samples (χ_1_ = 0.53, *p* = 0.47). The sample size was estimated based on the previous study applying the same procedure and same-different tasks in adults (Oldrati et al., 2020).

#### Stimuli and task

In Experiment 2, we used the same stimuli, trial structure and data handling of Experiment 1a. However, in this experiment participants were asked to perform two same-or-different judgment tasks focusing either on the emotion expression or on the sex of the stimuli. Participants indicated whether the emotion (emotion comparison task) or the sex (sex comparison task) of the central stimulus matched or not that of the stimuli at the sides.

#### Results on accuracy

Following data filtering, separate LMM were estimated for accuracy and RTs with TASK (emotion comparison vs. sex comparison), STIMULUS-TYPE (face vs. body), and TASK-IRRELEVANT CONGRUENCY (congruent vs. incongruent) and their 3-way interaction as fixed factors, and intercepts and SUBJECTS and STIMULUS IDENTITY (of the central stimulus) as random factors.

**Figure 4:**
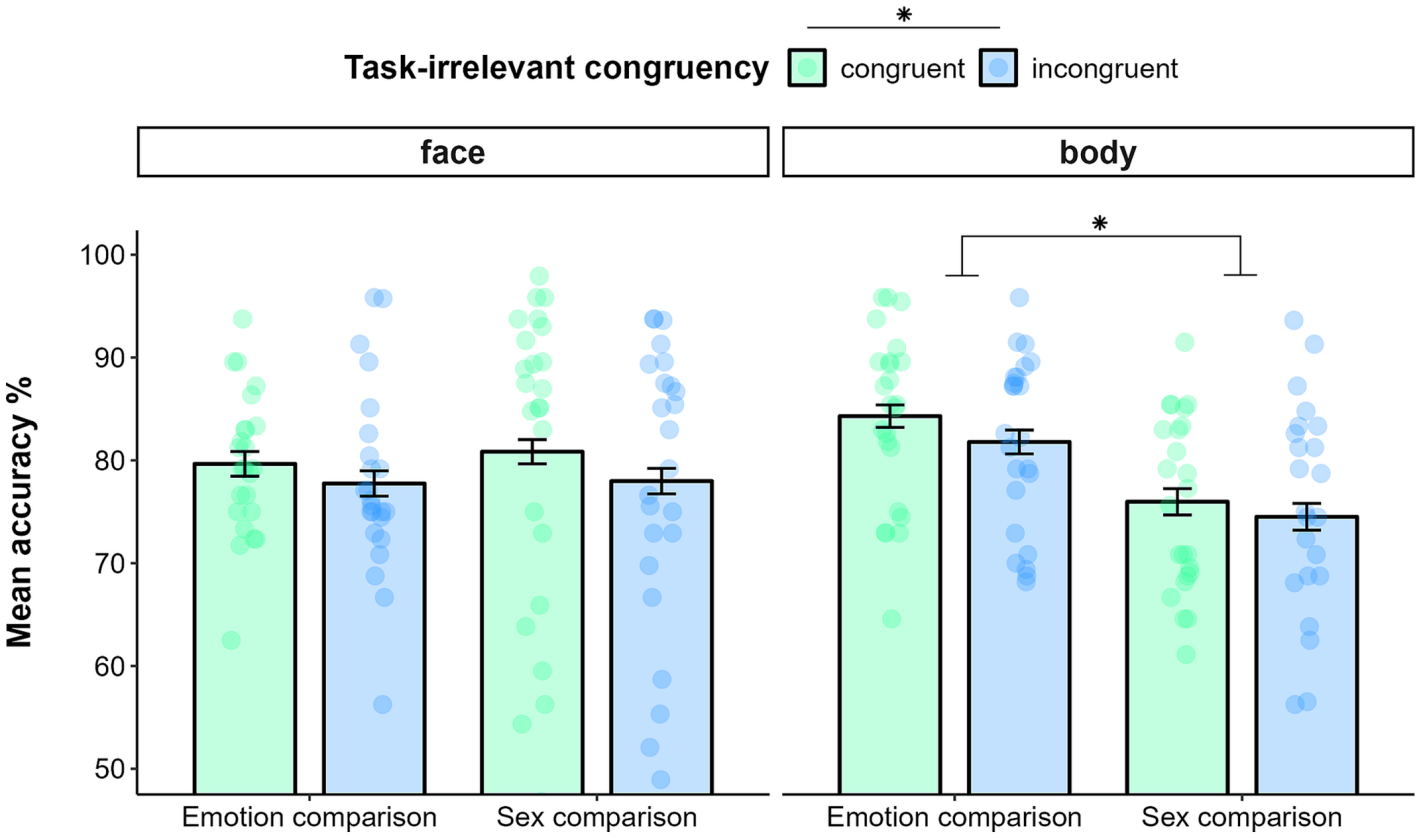
Mean accuracy in comparing faces (left panel) and bodies (right panel) as function of task (Emotion comparison vs. Sex comparison) and task-irrelevant congruency (sex congruency in emotion comparison; emotion congruency in sex comparison) in Experiment 2. Error bars represent ±1 SE. * *p* < 0.05.

Data filtering resulted in deleting 2.4% of trials in the emotion comparison task and 2.8% of trials in the sex comparison task. The GLMM on accuracy showed a main effect of TASK (χ_(1)_ = 17.58, *p* < 0.0001), indicating better performance for the emotion comparison (*M* = 0.81, SE = 0.01) than for the sex comparison task (*M* = 0.77, SE = 0.01). Then, the analysis yielded a significant main effect of TASK-IRRELEVANT CONGRUENCY (χ_(1)_ = 6.43, *p* = 0.01). Indeed, irrespectively of the task at hand and of the type of stimulus presented, participants were more accurate in responding when the task-irrelevant dimension was congruent (*M* = 0.80, SE = 0.01) than incongruent (M = 0.78, SE = 0.01). Furthermore, the model yielded a significant interaction effect of TASK* STIMULUS-TYPE (χ_(1)_ = 26.01, *p* < 0.0001). *Post hoc* analysis indicated that, while participants were more accurate in comparing the emotion (*M* = 0.83, SE = 0.01) than the sex of bodies (*M* = 0.75, SE = 0.02; *p* < 0.0001), such a difference did not emerge for faces (emotion: *M* = 0.79, SE = 0.01; sex: *M* = 0.79, SE = 0.02; *p* = 0.55; see Figure 4). The exploratory analysis confirmed the effects of the model described above. The effect of AGE and its interactions were non-significant (all *p* > 0.1).

### Results on RTs

The LMM on RTs showed a significant main effect of STIMULUS-TYPE (χ_(1)_ = 40.1, *p* < 0.0001), with participants being faster in comparing faces (*M* = 1473.38, SE = 31.79) than bodies (*M* = 1585.54, SE = 35.72). Additionally, a significant interaction effect of TASK* STIMULUS-TYPE emerged from the analysis (χ_(1)_ = 6.67, *p* < 0.01). This effect was driven by faster RTs during sex comparison (*M* = 1,568.16, SE = 51.58) than emotion comparison (*M* = 1,602.91, SE = 49.84) of bodies, but not of faces (emotion comparison: *M* = 1,456.65, SE = 44.78; sex comparison: *M* = 1,409.11, SE = 45.47). However, none of the two pairwise comparisons survived the application of the FDR correction (all *p* > 0.06). The exploratory analysis confirmed the main and interaction effects emerged in the model described above. Additionally, the analysis yielded a significant main effect of AGE (χ_(1)_ = 12.44, *p* < 0.001), indicating faster responses as the age of the participants increased. The analysis also yielded a significant interaction effect of AGE*STIMULUS-TYPE (χ_(1)_ = 5.34, *p* = 0.02). Even though the slopes of both faces (b = −67.3, CI: −111.0 − 23.8) and bodies (b = −79.5, CI: −123.0 − 36.1) were significant, indicating faster responses as age increased, a significant difference was detected between slopes (*p* = 0.02), describing a slightly greater acceleration with age in processing bodies than faces. No other effect was significant (all *p* > 0.08).

Accuracy and RTs among conditions in both tasks are reported in Table 2.

**Table 2:** Mean (SD) RTs and Accuracy for each experimental condition in Experiment 2.

	Emotion comparison				Sex comparison			
	Face		Body		Face		Body	
Task-irrelevant features	RTs	Acc	RTs	Acc	RTs	Acc	RTs	Acc
*Congruent*	1,457.7 (295.2)	0.80 (0.07)	1,613.9 (342.7)	0.84 (0.08)	1,472.8 (327.0)	0.81 (0.15)	1,573.6 (354.8)	0.76 (0.08)
*Incongruent*	1,455.6 (331.0)	0.78 (0.09)	1,591.9 (354.9)	0.82 (0.08)	1,507.4 (308.6)	0.78 (0.09)	1,562.7 (367.4)	0.75 (0.12)

#### Discussion experiment 2

The results indicated that broadening the focus of attention to include the lateral stimuli triggered a bottom-up interference of both emotional and non-emotional feature conflict (i.e., emotion and sex incongruence) on the main task, as expected. This experimental manipulation, by increasing the cognitive demand, may have disabled a proactive control relying on space-based selection of input (active in the Flanker paradigm), leading task-irrelevant cues, either emotional or sexual, to intrude on the processing of the targets in the task at hand. In other words, while participants were able to filter out the emotional features of the flankers when task-relevant in experiment 1a e 1b, possibly due to an efficient space-based filtering, they failed to do so when the task required a feature-based filtering mechanism. This result seems to confirm that the influence of emotion on cognitive processing is a highly conditional phenomenon, susceptible to methodological choices. As opposed to emotional cues, sexual cues demonstrated to be pervious to both filtering strategies, as suggested by the intrusion exerted by such dimension on attention in both experiment 1a and experiment 2; we interpreted this in light of a greater saliency of sexual features for children.

Conversely, our previous findings in adults showed a specific bottom-up intrusion of emotional features in the processing of sexual bodily cues, suggesting that feature-based mechanism were effective in filtering out only task-irrelevant non-emotional features of faces (Oldrati et al., 2020). We speculate that the present results may reflect a suboptimal (or not fully developed) feature-based filtering mechanism, which led both emotional and sexual cues to exert a bottom-up modulation of the processing of sexual and emotional features of the target, respectively.

### Experiment 3

In light of the results of Experiment 1, suggesting that sexual cues are able to affect the processing of emotional features, and of Experiment 2, confirming the bottom-up modulation of sex on emotion processing, we conducted a third experiment. The aim of this last experiment was to test whether sexual cues can affect the recognition of the sex of faces and bodies at an explicit level, by exerting a top-down modulation.

We administered a Flanker paradigm to examine the influence of sex cues, when task-relevant, on the allocation of attentional resources. This experiment was conceived to consolidate the results of Experiment 1a, in which, differently than in adults (Oldrati et al., 2020), sexual features seemed to modulate children’s and adolescents’ attention when both task-relevant and task-irrelevant in face and body blocks. Thus, we expected the congruency of the sex cue to modulate participants’ performance and the modulation to be comparable for faces and bodies. Furthermore, in this experiment, we increased sample size in order to test the effect of the participants’ age. We expected more adult-like performance in older participants, with less effect of the dimension of sex with increasing age.

#### Participants

One-hundred and ten healthy children and adolescents were recruited and participated in Experiment 3. The sample size was estimated based on the effect size of SEX CONGRUENCY emerged in experiment 1a. In more detail, we calculated the effect size of such effect considering only the emotion congruent trials, in order to align to the task of experiment 3, in which the emotion expression did not vary among the stimuli of the array. Using the G*power software (Faul et al., 2007), by setting the effect size Cohen’s *f* = 0.3 for a 2×2 within-subject ANCOVA with one covariate (age), with the alpha threshold of *p* = 0.05 and the desired power (1 - β) at 0.85, the computation indicated that a sample size of 102 was sufficient to detect the effects of interest. Expecting about 10% drop-offs, we recruited 110 participants. Among these, an inspection of accuracy scores signalled ten potential outliers. Two participants (1.8% of the total sample; 2 M, M = 10.5, SD = 3.54 years old) performed at chance level in both stimulus blocks (face and body) and were removed from analysis. Of other seven participants (6.4% of the total sample; 3 M / 4F, M = 9.14, SD = 1.57 years old), we included either the face or the body block, due to a chance-level performance in one of the two blocks. Thus, the final sample included one-hundred and eight participants (52 M / 56F, age range 8–16, M = 10.98, SD = 2.29 years old). In more detail, it included nineteen 8-years old, sixteen 9-years old, fifteen 10-years old, seven 11-years old, thirty-two 12-years old, three 13-years old, five 14-years old, six 15-years old and five 16-years old participants.

#### Stimuli and task

For this experiment, we used the same stimuli, trial structure and data handling of Experiment 1a. However, here participants performed only a sex recognition task. In separate blocks, they were required to discriminate the sex of the target face or body, while trying to ignore the flanker stimuli. However, differently from Experiment 1a, the congruency of emotional cues did not vary for the central and lateral stimuli; thus, the emotion of faces/bodies was always congruent (i.e., all happy or all fearful stimuli).

#### Results on accuracy

Following data filtering, separate LMM were estimated for accuracy and RTs with STIMULUS TYPE (face vs. body), SEX CONGRUENCY (congruent vs. incongruent), AGE and their 3-way interaction as fixed factors, and intercepts and SUBJECTS and STIMULUS IDENTITY (of the target) as random factors.

**Figure 5:**
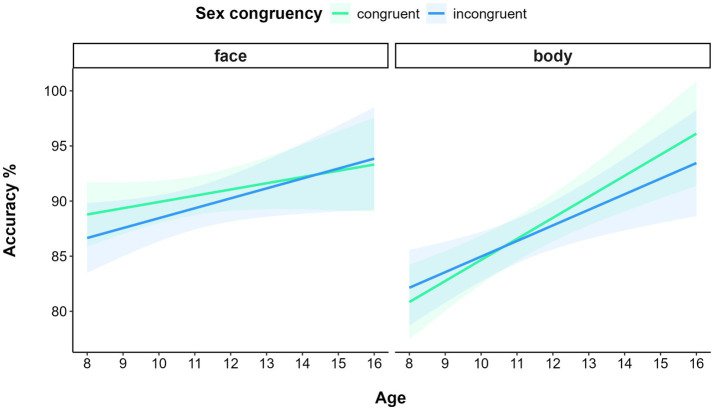
Relationship between Accuracy and Age (in years) for faces (left panel) and bodies (right panel) as a function of Sex congruency in Experiment 3. Shaded areas represent 95% confidence intervals.

Data filtering resulted in deleting 0.94% of trials. The GLMM on accuracy showed a significant main effect of STIMULUS TYPE (χ_(1)_ = 4.89, *p* = 0.03), with participants being more accurate in processing faces (M = 0.90, SE = 0.01) than bodies (M = 0.87, SE = 0.01). The model also yielded a significant main effect of AGE (χ_(1)_ = 11.58, *p* < 0.001): the older the participants, the more accurate their performance. The interaction STIMULUS TYPE* AGE was also significant (χ_(1)_ = 7.69, *p* = 0.01). In fact, although both the slopes of faces (b = 0.09, CI: 0.02–0.16) and bodies (b = 0.15, CI: 0.7–0.22) were significant, indicating better performance with increasing age, a significant difference was detected between slopes (*p* < 0.01), indicating a faster and larger improvement with age in processing bodies than faces. Moreover, the significant interaction of STIMULUS-TYPE* SEX CONGRUENCY* AGE (χ_(1)_ = 4.09, *p* = 0.04) further specified these interaction effects. A visual inspection of the 3-way interaction suggested an inversion of the sex congruency effect according to the type of stimuli with increasing age: a facilitation for sex congruent compared to sex incongruent face trials was present for younger participants, whereas older participants display a facilitation for sex congruent compared to sex incongruent body trials (Figure 5).

An exploratory analysis was carried out to confirm this effect by running a GLMM model with AGE operationalized as a categorical variable, obtained by dividing the sample into two subgroups. The “under 12” subgroup included 57 children under 12 years of age (M 23 / F 34, M = 9.18, SD = 1.04 years old), the “above 12” group included 51 preadolescents and adolescents above 12 years of age (M 29 / F 22, M = 13.0, SD = 1.47 years old). Alongside the main effects of STIMULUS-TYPE (χ_(1)_ = 4.91, *p* = 0.03) and AGE (χ_(1)_ = 9.98, *p* < 0.005), which also emerged in the model described above, the model confirmed the significant interaction effect of STIMULUS TYPE * SEX CONGRUENCY * AGE (χ_(1)_ = 4.78, p = 0.03). Pairwise comparison showed that the difference between sex congruent and incongruent trials in the “above 12” group was not significant for face stimuli (*p* = 0.5), but marginally significant for body stimuli (*p* = 0.06). Similarly, the difference between sex congruent and incongruent trials in the “below 12” group was not significant for body stimuli (*p* = 0.23), but marginally significant for face stimuli (*p* = 0.08).

#### Results on RTs

The LMM on RTs showed a significant main effect of STIMULUS-TYPE (χ_(1)_ = 21.92, *p* < 0.0001), with participants being faster in processing faces (*M* = 925.16, SE = 14.08) than bodies (*M* = 1005.75, SE = 16.92). The model also yielded a significant main effect of SEX-CONGRUENCY (χ_(1)_ = 4.49, p = 0.03), since participants were faster in recognizing the sex of the target in congruent (*M* = 954.12, SE = 19.43) than in incongruent trials (*M* = 965.97, SE = 20.25). Lastly, the main effect of AGE was significant (χ_(1)_ = 15.17, p < 0.0001): the older the participants, the faster their responses. Other effects were non-significant (all *p* > 0.09).

#### Discussion experiment 3

In this Flanker task, participants were asked to recognize the sex of a target stimulus while ignoring the distractors, whose sex could match or not with the target. The results suggested that, despite the participants had to focus on the central target and ignore the lateral flankers, the congruency between the sexual features of the target and flanker stimuli impacted on sex discrimination performance. In fact, participants were less accurate in recognizing the sex of the target if it did not match the sex of the flanker stimuli at the side. This result is in line with the significant main effect of sex congruency emerged in experiment 1a, suggesting that, in children and adolescents, sex cues may be more influential than the emotion expressions in guiding the processing of faces and bodies.

## General discussion

The processing of social-cues, like faces and bodies, is fundamental for our survival. However, their relevance for adapting in social environments may change across development. Here, we tested to what degrees attention modulated the processing of emotion and sex conveyed by faces or bodies in a sample of typically developing children and adolescents, applying a Flanker (Experiment 1a, 1b and 3) and a same-or-different judgment paradigm (Experiment 2). A first finding indicated the presence of an interaction between the emotional and sexual dimensions in the processing of bodies. In fact, exclusively in the Emotion recognition task, the congruency between the sex of the target and flankers facilitated the recognition of the emotion expressions of the target bodies in emotion incongruent trials. This result has been interpreted in light of the Garner interference effect, for which the attentional control system struggles to disregard sexual or emotional cues when the primary objective of the task at-hand is centered around emotion or sex, respectively (Gilboa-Schechtman et al., 2004; Atkinson et al., 2005; Becker, 2017; Craig and Lipp, 2023). In the context of this experiment, the aforementioned effect further qualified a significant main effect of sex congruency, which we interpreted as indicative of a potential greater saliency of sex, as compared to emotion, in guiding the processing of face and bodies in children and adolescents. This observation was further supported by the results of the same-or-different task in Experiment 2, which indicated that, disabling a space-based filtering strategy (which is active in the Flanker paradigm), not only task-irrelevant sexual, but also task-irrelevant emotional cues intruded on the processing of face and body targets. The emergence of a bottom-up influence of emotion was traced back to the increased cognitive load posed by this task, which may call for feature-based filtering mechanism (possibly immature in our sample) when screening the whole array of stimuli. Finally, the finding of a top-down modulation of attention control on the recognition of the sex of faces and bodies at an explicit level further supported our first interpretation about the saliency of sex as a social dimension for children and adolescents.

Going in more detail into the results, in the last experiment we observed a higher accuracy in processing faces than bodies, in line with previous evidence (Bank et al., 2015; Robbins and Coltheart, 2015). Moreover, we found a more pronounced improvement in the processing of bodies (emerged also in experiment 1b), driven by a facilitation of face over body processing in younger participants, which tended to disappear with increasing age. Accordingly, similar to the findings of developmental studies of face perception (Golarai et al., 2007), neuroimaging findings (Ross et al., 2014) suggested that the body-selective areas in the visual cortex – namely the fusiform body area (FBA) and the extrastriate body area (EBA) – do not become adult-like in terms of extent and strength of activation until the second decade of life, although they show similar sensitivity in both children and adults (Peelen et al., 2009; Pelphrey et al., 2009). This result differs to that of Bank et al. (2015), who observed a similar developmental trajectory for the memory of faces and bodies in 6–10 years old children. However, it is noteworthy that we examined a broader age-range and specifically tested the ability to recognize facial and bodily sexual features.

Furthermore, we reported a congruency effect of facial sexual features in younger participants, and *vice-versa* a congruency effect of bodily sexual features in older participants. We speculated that, in children, sex recognition is influenced by attention control due to the greater saliency that this dimension has when conveyed by facial features. Indeed, although children seem to improve in judging the sex of a face when devoid of superficial cues (e.g., make-up or hair) after age 7 (Wild et al., 2000), they also show differential behavioral response to male/female faces since early infancy (Quinn et al., 2002). The disappearing of such sex congruency effect with increasing age would reflect a fine-tuning of the mechanisms sub-serving the recognition of facial features. Thus, this could mean that, becoming more efficient, this process would become more automatic. This hypothesis is in keeping with evidence indicating that the improvement in processing faces (Seitz, 2002; Susilo et al., 2013; Fuhrmann et al., 2016) continues throughout childhood till adolescence, possibly coinciding with the maturation of the frontal lobe (Kolb et al., 1992) and general perceptual and memory development (Robbins and Coltheart, 2015).

Similarly, the emerging of the attentive control on sex recognition for body stimuli with increasing age could be due to an increased saliency of the sexual dimension of the body during adolescence. Indeed, although the ability to exploit sexual dimorphism to compel sex categorization of bodies develops already between the ages of 4 and 6 (Johnson et al., 2010), secondary sex characteristics of the body rise in importance by age 10 and increase with age in influencing judgment of attractiveness (Connolly et al., 2004; Sabiniewicz et al., 2015). During puberty, the child passes through identifiable stages of development of secondary sex characteristics, which need to be integrated into a new body image. Sex has been detected as a factor influencing body dissatisfaction among urban adolescents (Xanthopoulos et al., 2011). Accordingly, studies examining virtual self-representation found that the creation of avatars becomes more detailed, in terms of facial and bodily sexual features, as adolescents’ age increases (Villani et al., 2012, 2016). However, for what concerns the RTs, we found a main effect of sex congruency, but not its interaction with the age of the participants, indicating faster responses in recognizing the sex of the target regardless of whether it was a face or a body.

As hypothesized for faces, the absence of sex congruency effects in adults (Oldrati et al., 2020) would reflect, on one hand, a fine-tuning of the processing of bodies throughout adolescence and, on the other hand, an increase of informative value of emotion for adults. Indeed, in our previous study we found that emotion, but not sex, intruded in the main task, when either task relevant or task irrelevant. We speculated that, since emotion expressions reflect action intentions determined by their ultimate or proximate end (Ridderinkhof, 2017), emotion may be valued by adults as more informative about other social actors’ behavior as compared to sex. Conversely, social category schemas such as sex have been demonstrated to pose a great influence on children’s memory, attention and preferences (Ruble and Martin, 1998; Martin, 2000). For instance, children have been observed to remember better photographs of children engaging in gender-consistent than gender-inconsistent activities (Martin and Halverson, 1981), and to distort the recollection of gender-inconsistent information by changing the sex of target person associated to that information (Carter and Levy, 1988; Stangor and Ruble, 1989; Liben and Signorella, 1993). Accordingly, even for category-neutral information, sex has been demonstrated to play an important role in children’s initial encoding of others’ identity and behavior (Bennett and Sani, 2003), as they tend to spontaneously assimilate social information to gender-based categories (Bennett et al., 2000). Taken together, these pieces of evidence suggest that the categorization of sex can influence the further elaboration of other social information.

In line with this claim, Johnstone and Downing (2017), by applying a Gardner paradigm, found that irrelevant variation of sex interfered with weight judgments, whereas irrelevant variation of weight did not interfere with sex judgments. A possible interpretation of this asymmetrical pattern of interference points to a distinct processing of bodily features related to sex and weight, with an initial categorization of sexual features later on influencing the processing of weight. This asymmetrical influence between sex categorization and weight perception may hold importance for the understanding of anorexia nervosa, a condition characterized by stronger body-size distortion as well as greater body dissatisfaction (Mölbert et al., 2017). Interestingly, evidence of altered gray matter density of EBA, negatively correlating with body size misjudgments, has been observed in women with anorexia nervosa (Suchan et al., 2010). Further, in these patients, reduced connectivity between EBA – involved in processing of body parts and body size (Moayedi et al., 2021) – and the FBA – involved in processing whole body forms (Taylor et al., 2007) – was observed during body image perception and body size estimation tasks (Suchan et al., 2013). Hence, monitoring the developmental trend of the saliency of facial, but especially of bodily cues, to the perception of others’ sex in adolescence may provide cues for the understanding of clinical conditions involving body-size distortion and body dissatisfaction.

The conclusions that can be drawn from this study must be considered in light of a few limitations. To begin with, we included in the first three experiments (experiment 1a, 1b and 2) a broad age range (8–16 years old). Since the developmental changes occurring from childhood to adolescence do influence both cognitive and emotional processing (Larsen and Luna, 2018), including individuals of mixed age could have impact our results. Nevertheless, the exploratory analyses (in experiment 1b and 2) showed no significant interaction between age and the processing of emotion or sexual cues congruency. Further, the influence of age was directly addressed in experiment 3. Another potential limitation is that we did not control for the potential impact of the level of arousal of the stimuli, which was not assessed in this study. However, previous studies have provided validation data on arousal and valence of both face (Adolph and Alpers, 2010) and body (Borgomaneri et al., 2012) stimuli. Moreover, the choice of using flankers of the same identity could have facilitated the processing of the target stimulus due to a pop-out effect. However, there is evidence demonstrating that identity does not significantly distract from evaluating emotions in target and flanker (Mueller and Kuchinke, 2016). Yet, to the best of our knowledge, the effect of identity has not been assessed in flanker task probing sex recognition or for bodily stimuli.

To conclude, as pointed out about the interplay between attention control and emotion processing, we cannot rule out that the effects identified here could have been influenced by the type of stimuli selected or by the applied paradigms. Based on these possible limitations, we recognize that the interpretations of the present findings are open to challenge and that future research is needed to further support, or disconfirm, the interpretations provided. Nevertheless, the results point to developmental changes of the saliency of emotional and sex dimensions of faces and bodies, which should be taken into accounts when extending results from adult populations to the study of typically developing children and adolescents as well as to the evaluation and treatment of neuropsychiatric disorders.

## Data availability statement

The datasets presented in this study can be found in online repositories. The names of the repository/repositories and accession number(s) can be found at: Zenodo repository, 10.5281/zenodo.8432107.

## Ethics statement

The studies involving humans were approved by Commissione di Garanzia per il rispetto dei principi etici nell’attività di ricerca sugli esseri umani/Institutional Review Board (C.G.P.E.R./I.R.B.). The studies were conducted in accordance with the local legislation and institutional requirements. Written informed consent for participation in this study was provided by the participants' legal guardians/next of kin.

## Author contributions

VO: Conceptualization, Data curation, Formal analysis, Investigation, Writing – original draft, Writing – review & editing. AB: Conceptualization, Funding acquisition, Writing – review & editing. GP: Conceptualization, Writing – review & editing. CU: Conceptualization, Formal analysis, Funding acquisition, Supervision, Writing – review & editing.

## Footnotes

^1^e.g. https://orwh.od.nih.gov/sex-gender
